# Reply to the letter of Groenveld et al.: ‘Routine measurement of oesophageal temperature during cryoballoon pulmonary vein isolation’

**DOI:** 10.1007/s12471-021-01558-7

**Published:** 2021-03-11

**Authors:** J. M. van Opstal, Y. J. Stevenhagen, P. F. H. M. van Dessel, M. F. Scholten

**Affiliations:** Department of Cardiology, Thorax Centrum Twente, Medisch Spectrum Twente, Enschede, The Netherlands

To the Editor,

We would like to thank Groenveld et al. for this opportunity to discuss the results of our study regarding the high incidence of (ultra)low oesophageal temperatures during cryoballoon pulmonary vein isolation (PVI) and our recommendation to routinely measure oesophageal temperatures to avoid atrio-oesophageal complications during PVI [1]. Groenveld et al. suggest that routine oesophageal temperature measurement during PVI is unnecessary as the incidence of atrio-oesophageal fistula is low and most oesophageal lesions are asymptomatic. Furthermore, they argue that measurement of oesophageal temperatures cannot prevent oesophageal lesions [2, 3].

Although the incidence of atrio-oesophageal fistula is low, it is often a deadly complication of treatment for a nonfatal arrhythmia. Furthermore, the incidence of gastroparesis and hypomotility (caused by damage to the branches of the vagal nerve surrounding the oesophagus) is much higher (up to 17%) [4]. As such, oesophageal lesions should be avoided as they are most likely the precursor of these dreadful complications.

In line with this, the 2017 Heart Rhythm Society/European Heart Rhythm Association expert consensus statement on catheter and surgical ablation of atrial fibrillation states it is reasonable to use an oesophageal temperature probe during ablation procedures to monitor oesophageal temperatures to help guide energy delivery (Class IIa) [5]. Luminal oesophageal temperature—guided PVI significantly reduces the incidence of thermal oesophageal lesions [6]. Moreover, although lesions cannot always be avoided, they tend to be smaller in patients when using a temperature probe [7].

The most probable reason that oesophageal lesions are still present despite measurement of luminal oesophageal temperatures is that current temperature probes do not provide adequate coverage of the oesophageal width. As a result, the temperature of the oesophageal tissue at risk—situated closest to the posterior wall of the left atrium—is not always sufficiently monitored.

Therefore, further research and development in this field is necessary to eliminate PVI-related atrio-oesophageal complications. Until that time, we have to use the oesophageal thermoprobes at our disposal, bearing in mind Hippocrates’s phrase *primum non nocere* (first, do no harm).
